# Role of Transient Receptor Potential Ankyrin 1 Ion Channel and Somatostatin sst4 Receptor in the Antinociceptive and Anti-inflammatory Effects of Sodium Polysulfide and Dimethyl Trisulfide

**DOI:** 10.3389/fendo.2018.00055

**Published:** 2018-02-27

**Authors:** István Z. Bátai, Ádám Horváth, Erika Pintér, Zsuzsanna Helyes, Gábor Pozsgai

**Affiliations:** ^1^Department of Pharmacology and Pharmacotherapy, Medical School, University of Pécs, Pécs, Hungary

**Keywords:** transient receptor potential ankyrin 1, sst4, somatostatin, dimethyl trisulfide, polysulfide, carrageenan, luminol, IR-676

## Abstract

Transient receptor potential ankyrin 1 (TRPA1) non-selective ligand-gated cation channels are mostly expressed in primary sensory neurons. Polysulfides (POLYs) are Janus-faced substances interacting with numerous target proteins and associated with both protective and detrimental processes. Activation of TRPA1 in sensory neurons, consequent somatostatin (SOM) liberation and action on sst4 receptors have recently emerged as mediators of the antinociceptive effect of organic trisulfide dimethyl trisulfide (DMTS). In the frame of the present study, we set out to compare the participation of this mechanism in antinociceptive and anti-inflammatory effects of inorganic sodium POLY and DMTS in carrageenan-evoked hind-paw inflammation. Inflammation of murine hind paws was induced by intraplantar injection of carrageenan (3% in 30 µL saline). Animals were treated intraperitoneally with POLY (17 µmol/kg) or DMTS (250 µmol/kg) or their respective vehicles 30 min prior paw challenge and six times afterward every 60 min. Mechanical pain threshold and swelling of the paws were measured by dynamic plantar aesthesiometry and plethysmometry at 2, 4, and 6 h after initiation of inflammation. Myeloperoxidase (MPO) activity in the hind paws were detected 6 h after challenge by luminescent imaging. Mice genetically lacking TRPA1 ion channels, sst4 receptors and their wild-type counterparts were used to examine the participation of these proteins in POLY and DMTS effects. POLY counteracted carrageenan-evoked mechanical hyperalgesia in a TRPA1 and sst4 receptor-dependent manner. POLY did not influence paw swelling and MPO activity. DMTS ameliorated all examined inflammatory parameters. Mitigation of mechanical hyperalgesia and paw swelling by DMTS were mediated through sst4 receptors. These effects were present in TRPA1 knockout animals, too. DMTS inhibited MPO activity with no participation of the sensory neuron–SOM axis. While antinociceptive effects of POLY are transmitted by activation of peptidergic nerves *via* TRPA1, release of SOM and its effect on sst4 receptors, those of DMTS partially rely on SOM release triggered by other routes. SOM is responsible for the inhibition of paw swelling by DMTS, but TRPA1 does not contribute to its release. Modulation of MPO activity by DMTS is independent of TRPA1 and sst4.

## Introduction

Inorganic polysulfides (POLYs; hydrogen polysulfide) have been demonstrated to be synthesized in the human body (1). These species possess antioxidant and radical scavenging properties. Beside *in vitro* systems, these findings were confirmed in lung tissue from patients suffering from chronic obstructive pulmonary disease too (2–5). According to some opinions inorganic POLYs might mediate persulfidation of cysteine residues of proteins, a process traditionally attributed to hydrogen sulfide (H_2_S) (6). Dimethyl trisulfide (DMTS) is an organic trisulfide compound naturally occurring in garlic. It is used widely as a food additive (7). Recently, DMTS has been patented in the US as a parenteral antidote of cyanide poisoning (8). This adds vastly to the translation potential of the drug. We have reported lately antinociceptive properties of DMTS against mechanical hyperalgesia evoked by heat injury in mice. Transient receptor potential ankyrin 1 (TRPA1) ion channels and somatostatin (SOM) sst4 receptors contribute pivotally to these effects (9). Chemically, alkyl trisulfides (such as DMTS) produce tri/disulfide metabolites with the thiol groups of cysteine amino acids (unlike inorganic POLYs leading to protein persulfidation). Others propose organic trisulfides to be sources of hydrogen sulfide (H_2_S) (10). Based on the latest findings, H_2_S in concert with nitric oxide reacts with thiol residues of proteins (11, 12). H_2_S released from organic trisulfides might influence protein-associated metal atoms too (13). Organic trisulfides were reported to exert antioxidant and anti-inflammatory effects mostly studied in animal models of inflammatory bowel disease (14–16). Inorganic POLYs are known to interact with functional cysteines of the TRPA1 ion channel (17). As mentioned above our previous work suggests that one of the targets of DMTS is the ion channel TRPA1 as well (9).

Transient receptor potential ankyrin 1 is a non-selective cation channel permeable to Ca^2+^ and Na^+^. TRPA1 is a member of transient receptor potential ankyrin subfamily of ion channels, itself being a subdivision of the transient receptor potential family. TRPA1 is the only ankyrin-type TRP channel to be found in mammals. Polymodal TRPA1 channels might be opened by chemical substances, temperature, mechanical stimuli, potential difference, or changes of pH. Electrophilic agents—most probably including organic trisulfide compounds—excite TRPA1 by forming covalent bonds with cysteine residues (18). TRPA1 is mostly expressed in primary nociceptor neurons, but it was evinced in the cornea, skin, pancreas, spleen, lung, kidney, testis, and the human endometrium (19). Expression of TRPA1 channels in polymorphonuclear granulocytes of patients suffering from chronic inflammatory disease was shown to correlate with nociception (20). The role of TRPA1 is known in complete Freund’s adjuvant-induced inflammation. However, no involvement was detected in carrageenan-evoked paw inflammation (21, 22). TRPA1 channels are typically expressed by sensory neurons containing neuropeptides (e.g., SOM). Activation of the channel leads to Ca^2+^ influx into the nerve endings and release of peptides. Earlier we found SOM liberation from murine sensory neurons upon stimulation with DMTS (9).

Somatostatin is a cyclic peptide with important endocrine function besides its presence in the sensory nervous system (23). SOM is expressed in 17.8% of human dorsal root ganglion neurons. The peptide might be liberated by TRPA1 agonists (24). Unlike most neuropeptides, SOM is distributed by the bloodstream and exerts antinociceptive and anti-inflammatory effects distant from the release site in numerous animal models of inflammatory disease (25). These could be ameliorated by depletion of peptides from sensory nerves, administration of anti-SOM antibody or SOM receptor antagonist (24). According to previous data, these effects are mediated by one of five SOM receptors: sst4 (9, 26–29). Antinociceptive and anti-inflammatory effects could be mimicked by two different agonists (TT-232, J-2156) of sst4 receptors. The agonists were ineffective in animals lacking the corresponding functional receptor (24, 30). Sst4 is present in sensory neurons, lymphocytes, and vascular endothelial cells enabling the transmission of the aforementioned beneficial effects of SOM (25).

In the present study, we set out to investigate the effect of inorganic sodium POLY and DMTS on the sensory-SOM-sst4 system in carrageenan-induced hind paw inflammation in genetically engineered mice lacking either functional TRPA1 or sst4. Both mechanical nociception and inflammatory parameters, such as paw swelling and myeloperoxidase (MPO) activity of accumulated neutrophil granulocytes, were assessed.

## Materials and Methods

### Animals

Experiments were conducted on genetically modified male mice lacking functional TRPA1 or sst4 receptors (KO) and their wild-type counterparts (WT; 2–4 months, 20–25 g) (27, 31). Age-matched animals were used in the study. The original heterozygous TRPA1 breeding pair was a generous gift from Pierangelo Geppetti (University of Florence, Italy). These mice were originally generated and characterized by Bautista and colleagues (31). Neither the strain with genetic modification of TRPA1 nor that with modified sst4 gene is available commercially. TRPA1 and sst4 WT and KO breeding lines were produced by crossing respective heterozygote animals. WT and KO animals were chosen from the resulting litter and used for further breeding (i.e., WT mice were mated with WT ones and KO mice with KO ones). For the fifth-generation clean WT and KO breeding lines were established and maintained by inbreeding. All animals were genotyped until generation 5 and random sentinel litters of the WT and KO lines afterward. Due to poor breeding performance of the sst4 colony, heterozygotes were used in the breeding even after the fifth generation and all offspring were genotyped for an extended period of time. Animals were bred and kept in the Laboratory Animal Centre of University of Pécs under standard pathogen free conditions at 24–25°C, 12 h light/dark cycles. Mice were housed in groups of 5–10 in polycarbonate cages (330 cm^2^ floor space, 12 cm height) on wood shavings bedding. Animals were provided standard diet and water *ad libitum*. All experimental procedures were carried out according to the European Communities Council Directive of 2010/63/EU. The studies were approved by the Ethics Committee on Animal Research, University of Pécs (license number: BA02/2000-47/2017).

### Carrageenan-Induced Hind Paw Inflammation

Inflammation of one hind paw was triggered by intraplantar injection of carrageenan (20 µL, 3% in saline). The contralateral paw received saline. The side of carrageenan injection was randomized. Animals were treated with either POLY (17 µmol/kg, i.p.) or DMTS (250 µmol/kg, i.p.) or the respective vehicle 30 min before challenge of the paws and every 60 min afterward (seven times altogether). POLY was prepared freshly before each application. DMTS was prepared daily.

### Preparation of POLY and DMTS Solutions

Polysulfide was prepared as described earlier (32). Stock solutions of hypochlorous acid and sodium sulfide nonahydrate were prepared in distilled water using polypropylene tubes blown with nitrogen gas beforehand. All later dilutions and reactions were performed in similar tubes. Reagents were kept on ice. Concentration of hypochlorous acid was calculated from the light extinction of the solution at 292 nm wavelength (E_292_ = 350 M^−1^cm^−1^). Concentration of sulfide was derived from the extinction at 230 nm (E_230_ = 7700 M^−1^cm^−1^) and the reaction with 5,5′-dithiobis(2-nitrobenzoic acid) (DTNB). Extinction of the reaction product of sulfide and DTNB was measured at 412 nm (E_412_ = 28,200 M^−1^cm^−1^). Sulfide concentration was calculated as the mean of the two values yielded by direct spectrophotometry and reaction with DTNB. Stock solutions of hypochlorous acid and sulfide were prepared daily. Sulfide stock solution was diluted further in distilled water to 60 mM. Hypochlorous acid solution was added slowly under stirring to produce 20 mM in the final volume. The reaction of sulfide and hypochlorous acid produces POLY. This POLY solution was diluted to twofold in distilled water containing 4.17% v/v 10x concentrated phosphate-buffered saline (PBS, pH 7.4). This amount of PBS renders the POLY solution isosmotic. Concentrated hydrochloric acid was then added by 5 µL under stirring to set the pH to 7.4 (app. 25–30 µL, as required). Concentration of the resulting POLY solution was measured by cold cyanolysis, as described earlier (33). Shortly, the isosmotic and isohydric POLY solution was alkalized by the addition of NH_4_OH and reacted with KCN. After 25 min incubation at room temperature formaldehyde and Goldstein reagent (FeCl_3_ dissolved in 18.38% HNO_3_) were added. Absorbance of the formed orange product was detected after 15 min reaction time at room temperature at 460 nm. POLY concentration was calculated using a standard curve constructed with KSCN. The buffered solution was found to contain 3.3 mM POLY, yielding a dose of 17 µmol/kg at 5 mL/kg. Isosmotic and isohydric POLY solution was injected into the mice immediately after production. PBS was used as vehicle control.

A DMTS solution of 1 M was prepared in dimethyl sulfoxide (DMSO). This solution was diluted to 100 mM in saline containing 2% v/v polysorbate 80. After slow dissolution, a further dilution commenced in saline to 25 mM. The 25 mM solution was injected at 10 mL/kg i.p. resulting in a dose of 250 µmol/kg. In vehicle, DMSO was applied instead of 1 M DMTS solution. Final DMTS solutions contained 2.24% v/v DMSO and 0.45% v/v polysorbate 80. Vehicle had 2.5% v/v DMSO.

### Measurement of Mechanical Pain Threshold of the Hind Paws

Mechanical hyperalgesia evoked by carrageenan was assessed by dynamic plantar aesthesiometry (DPA, Hugo Basile, Italy) 2, 4, and 6 h after the initiation of inflammation. Baseline values were taken on three separate days before paw challenge. Stimulator of the instrument reached 10 g “force” in 4 s.

### Detection of Paw Swelling by Plethysmometry

Swelling of inflamed and control hind paws was measured by plethysmometry (Hugo Basile, Italy). These measurements were performed following DPA experiments to prevent stressing the animals before aesthesiometry. Control measurements were conducted right after control DPA experiments on three separate days preceding paw challenge. Paw volumes were measured in cm^3^.

### Detection of MPO Activity in the Hind Paws by Luminescent Imaging

Animals were anesthetized with ketamine and xylazine (120 and 12 mg/kg) 6 h after hind paw challenge. Mice were injected i.p. with sodium luminol (5-amino-2,3-dihydro-1,4-phthalazine-dione; 150 mg/kg) dissolved in sterile PBS. Luminol signals reactive oxygen species correlated with MPO activity of neutrophil granulocytes *via* luminescence (34). Bioluminescence of luminol was captured 10 min after administration. Measurements were conducted in an IVIS Lumina II (PerkinElmer, Waltham, USA; 120 s acquisition, F/stop = 1, Binning = 8) instrument and Living Image^®^ software (Perkin-Elmer, Waltham, USA). Identical regions of interest (ROIs) were applied to both hind paws and calibrated units of luminescence (total radiance = total photon flux/s) originating from the ROIs were detected (35).

### Chemicals

All chemicals were purchased from Sigma Aldrich, Hungary unless otherwise stated. DMSO was from Reanal, Hungary. Ketamine was from Richter Gedeon, Hungary. Xylazine was from Eurovet Animal Health BV, Netherlands.

### Statistics

Data are presented as mean ± SEM. Two-way repeated-measure ANOVA followed by Bonferroni’s test was used for mechanonociceptive threshold and paw volume data. Some data on mechanonociceptive threshold were analyzed by plain one-way ANOVA followed by Tukey’s test. Results on MPO activity were analyzed by plain one-way ANOVA and Bonferroni’s test. Statistical analysis was performed by GraphPad Prism 5 software.

## Results

### Inhibition of Carrageenan-Evoked Mechanical Pain by POLY Is TRPA1 and sst_4_ Receptor-Dependent

**Figure 1:**
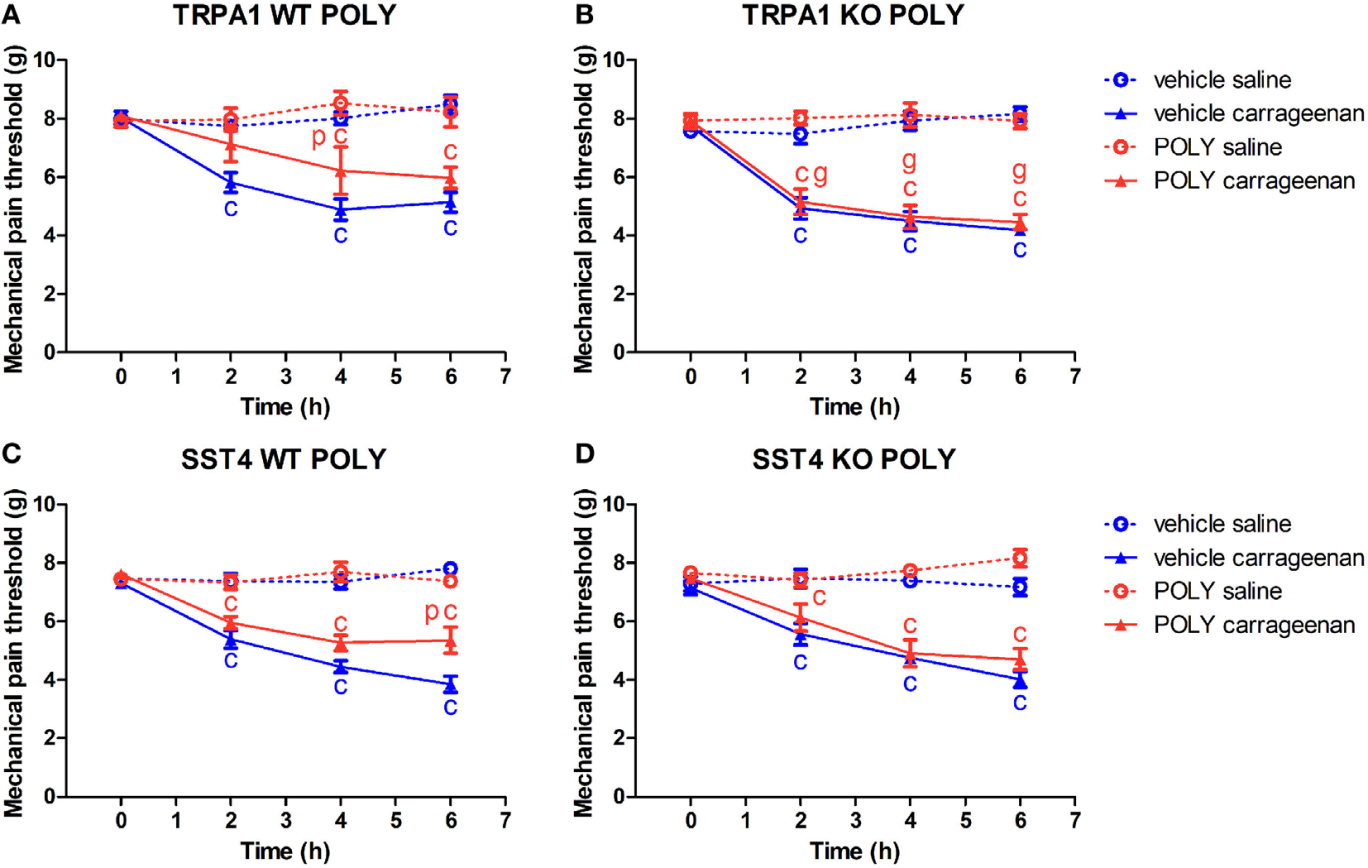
Antinociceptive effect of sodium polysulfide (POLY, 17 µmol/kg) in carrageenan-induced paw inflammation is mediated by transient receptor potential ankyrin 1 (TRPA1) and sst4 receptors. Mechanical pain threshold of saline or carrageenan-injected (3% in 20 µL saline) hind paws of **(A)** TRPA1 WT, **(B)** TRPA1 KO, **(C)** sst4 receptor WT, and **(D)** sst4 receptor KO animals in response to POLY or vehicle treatment. Data are shown as mean ± SEM. *n* = 6–8. ^c^*p* < 0.05 vs. saline-injected paws. ^p^*p* < 0.05 vs. vehicle of POLY. ^g^*p* < 0.05 vs. TRPA1 WT animals. Two-way repeated-measure ANOVA followed by Bonferroni’s multiple comparison test.

Carrageenan-injected paws of TRPA1 WT and KO mice undergoing vehicle administration developed significantly lowered mechanical pain threshold compared to saline-treated ones (*n* = 6–7; Figures 1A,B). POLY significantly reduced mechanical hyperalgesia in carrageenan-injected feet of TRPA1 WT animals in comparison with those of vehicle-treated ones (4.89 ± 0.36 vs. 6.22 ± 0.81 g at 4 h after challenge; *n* = 6–7; Figure 1A). Inhibitory effect of POLY on mechanical nociception in carrageenan-treated hind paws was lacking in TRPA1 KO animals compared to WT ones (7.12 ± 0.6 vs. 5.16 ± 0.44 g, 6.22 ± 0.81 vs. 4.64 ± 0.4 g, 5.97 ± 0.37 vs. 4.46 vs. 0.26 g at 2, 4 and 6 h after challenge; *n* = 6–7; Figure 1B). POLY had no effect on the mechanical pain thresholds of saline-injected feet of TRPA1 WT and KO animals (Figures 1A,B).

Similar to the above, both sst4 receptor WT and KO animals treated with the vehicle of POLY responded with reduced mechanical pain threshold to carrageenan administration (*n* = 6–8; Figures 1C,D). POLY significantly relieved mechanical nociception 6 h after challenge in carrageenan-injected feet of sst4 WT animals compared to those of vehicle-treated ones (3.85 ± 0.27 vs. 5.35 ± 0.45 g at 6 h after challenge; *n* = 7–8; Figure 1C). No effect of POLY was observed in sst4 KO mice. POLY did not affect mechanical pain thresholds of saline-treated paws of sst4 receptor WT and KO animals (Figures 1C,D).

### No Exclusive Role of TRPA1 Ion Channel in the Protective Effect of DMTS in Carrageenan-Induced Mechanical Hyperalgesia

**Figure 2:**
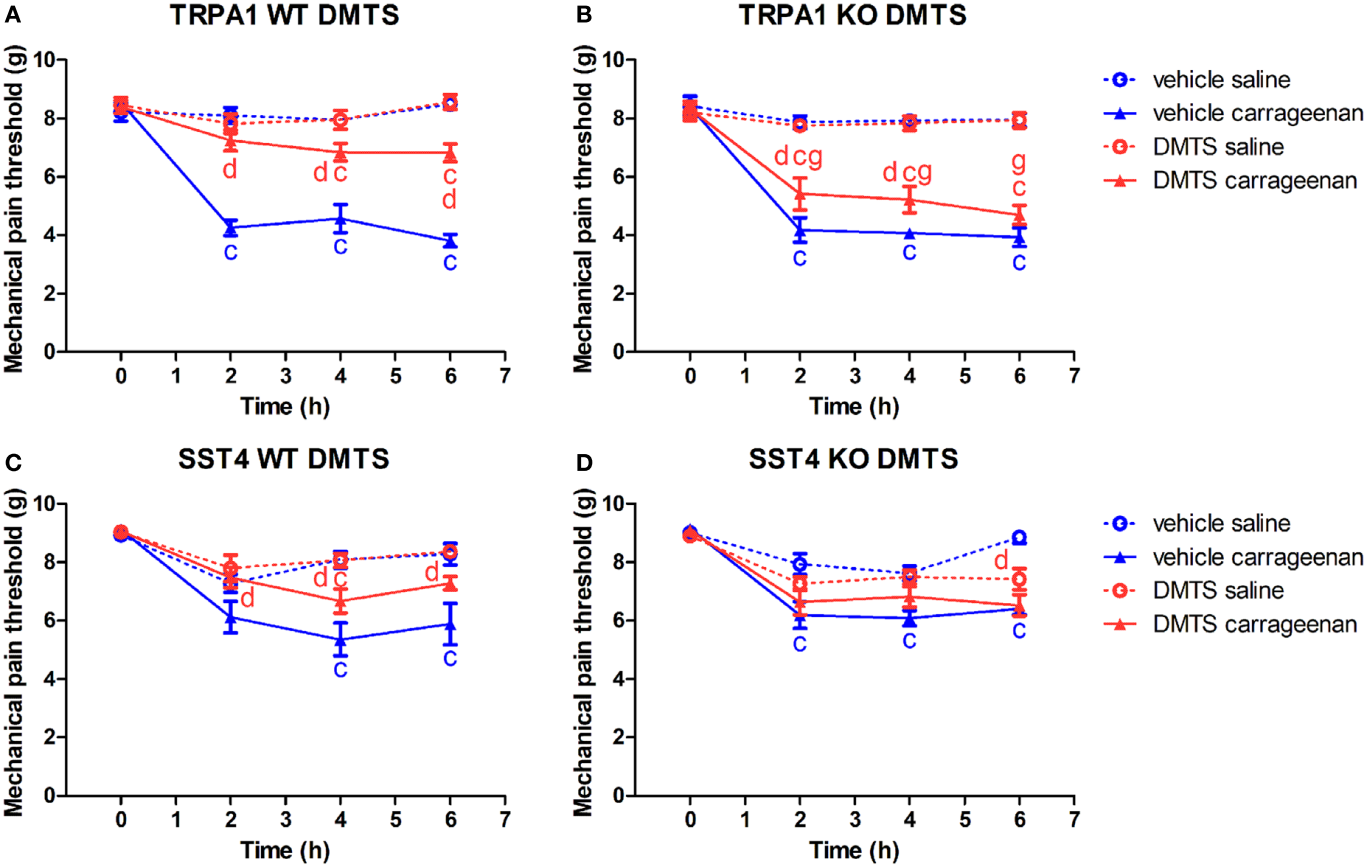
Antinociceptive effect of dimethyl trisulfide (DMTS, 250 µmol/kg) in carrageenan-evoked paw inflammation is independent of the transient receptor potential ankyrin 1 (TRPA1) ion channel, but is mediated by somatostatin (SOM) sst4 receptors. Effect of DMTS or vehicle treatment on mechanical pain threshold of either saline or carrageenan-treated (3% in 20 µL saline) hind paws of **(A)** TRPA1 WT, **(B)** TRPA1 KO, **(C)** sst4 receptor WT, and **(D)** sst4 receptor KO mice. Data are shown as mean ± SEM. *n* = 6–8. ^c^*p* < 0.05 vs. saline-injected paws. ^d^*p* < 0.05 vs. vehicle of DMTS. ^g^*p* < 0.05 vs. TRPA1 WT animals. Two-way repeated-measure ANOVA followed by Bonferroni’s multiple comparison test.

Carrageenan-injected hind paws of TRPA1 WT and KO animals treated with vehicle of DMTS developed mechanical hyperalgesia compared to saline-injected contralateral paws (*n* = 6–7; Figures 2A,B). Carrageenan-treated hind paws of TRPA1 WT mice undergoing DMTS administration showed significantly less hyperalgesia than those administered vehicle (*n* = 6–7; Figure 2A). Protective effect of DMTS was reduced in carrageenan-injected feet of TRPA1 KO animals compared to those of TRPA1 WT ones (*n* = 6–7; Figure 2B). However, DMTS still alleviated mechanical hyperalgesia in carrageenan-treated feet of TRPA1 KO mice at 2 and 4 h after challenge in comparison with vehicle-treated animals (*n* = 7; Figure 2B). Saline-injected paws of DMTS and vehicle-treated TRPA1 WT and KO animals did not differ from one another (Figures 2A,B).

Carrageenan-injected hind paws of sst4 receptor WT and KO animals being administered vehicle of DMTS exhibited mechanical hyperalgesia compared to saline-injected control feet (*n* = 7–8; Figures 2C,D). Carrageenan-treated hind paws of sst4 receptor WT mice injected with DMTS developed significantly smaller hyperalgesia than those of vehicle-treated control animals (*n* = 7; Figure 2C). Mechanical pain threshold of saline-treated paws of DMTS and vehicle-injected sst4 receptor WT animals did not differ statistically (Figure 1C). DMTS did not inhibit nociception in carrageenan-treated feet of sst4 receptor KO animals compared to those of their WT counterparts (Figure 2D). Saline-treated feet of vehicle-injected sst4 receptor KO animals developed significantly larger mechanical pain threshold at 6 h than those of DMTS-treated ones (*n* = 7–8; Figure 1D).

### POLY Does Not Affect Paw Swelling Evoked by Carrageenan

**Figure 3:**
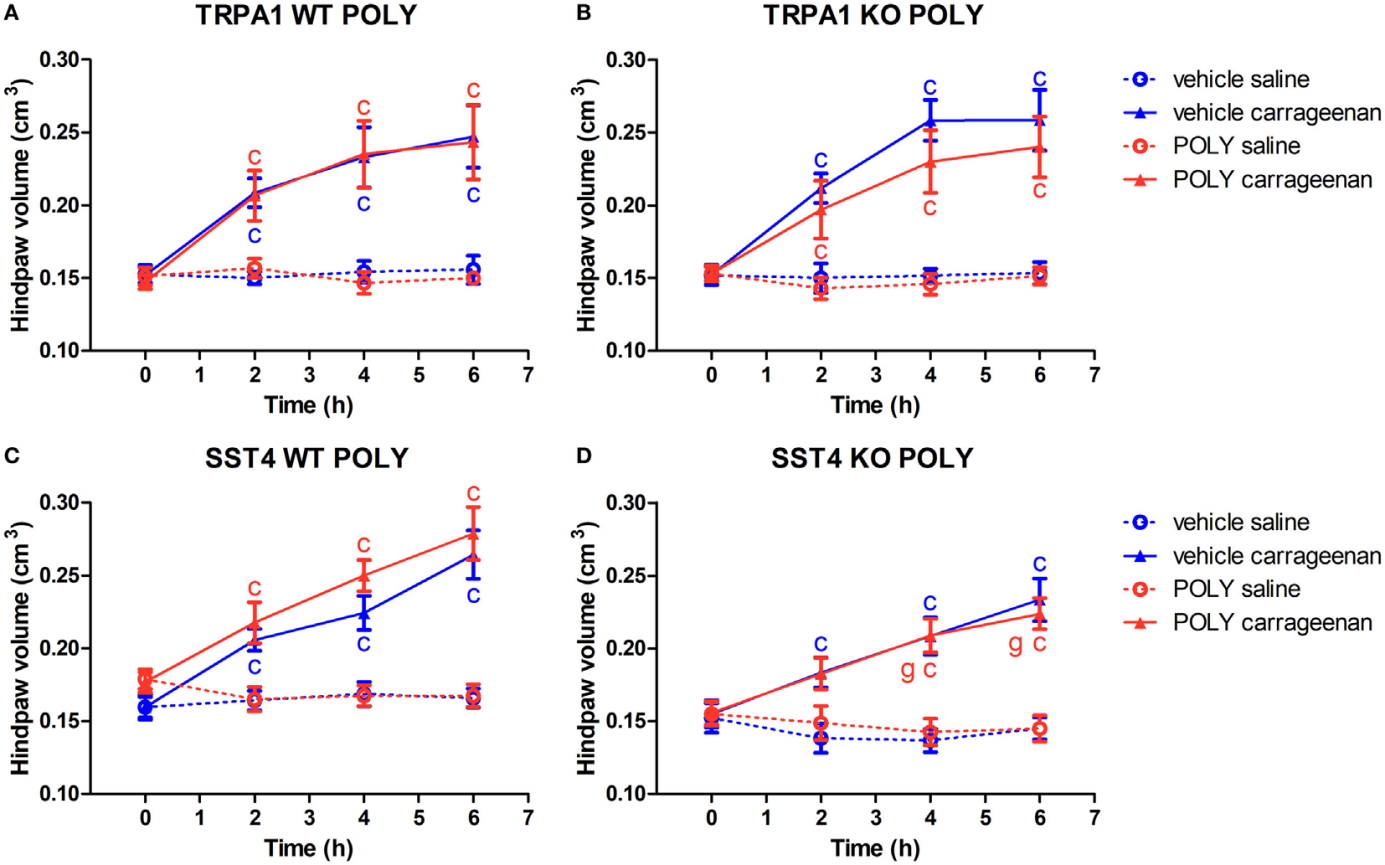
Sodium polysulfide (POLY; 17 µmol/kg, i.p.) does not affect paw swelling detected by plethysmometry in carrageenan-induced hind paw inflammation. Effect of POLY or vehicle treatment on paw swelling of either saline or carrageenan-treated (3% in 20 µL saline) hind paws of **(A)** transient receptor potential ankyrin 1 (TRPA1) WT, **(B)** TRPA1 KO, **(C)** sst4 receptor WT, and **(D)** sst4 receptor KO mice. Data are shown as mean ± SEM. *n* = 6–8. ^c^*p* < 0.05 vs. saline-injected paws. Two-way repeated-measure ANOVA followed by Bonferroni’s multiple comparison test.

Both vehicle and POLY-treated TRPA1 WT and KO mice exhibited significant paw swelling upon carrageenan stimulation of the hind paws. POLY had no statistically significant inhibitory effect on the swelling of the feet in TRPA1 WT or KO animals. T-values of two-way ANOVA followed by Bonferroni’s test for the comparison of POLY- and vehicle-treated carrageenan-injected paws of TRPA1 KO animals are the following: 0 h, 0.04846; 2 h, 0.8061; 4 h, 1.573; and 6 h, 1.018. A trend for inhibition by POLY can be seen in carrageenan-injected feet of TRPA1 KO mice in comparison to those of vehicle-treated ones that does not reach the level of statistical significance (*n* = 6–7; Figures 3A,B). POLY or vehicle treatment did not change paw volumes of saline-injected control paws. Similar results were obtained in sst4 receptor WT and KO mice regarding lack of statistically significant effect of POLY in either saline or carrageenan-injected paws compared to vehicle (*n* = 6–8). Volume of carrageenan-injected hind feet of sst4 KO mice was significantly smaller at 4 and 6 h post challenge than those of WT ones (*n* = 8; Figures 3C,D).

### Protective Effect of DMTS in Carrageenan-Evoked Paw Swelling Is Independent of TRPA1, but Is Mediated Through sst4 Receptors

**Figure 4:**
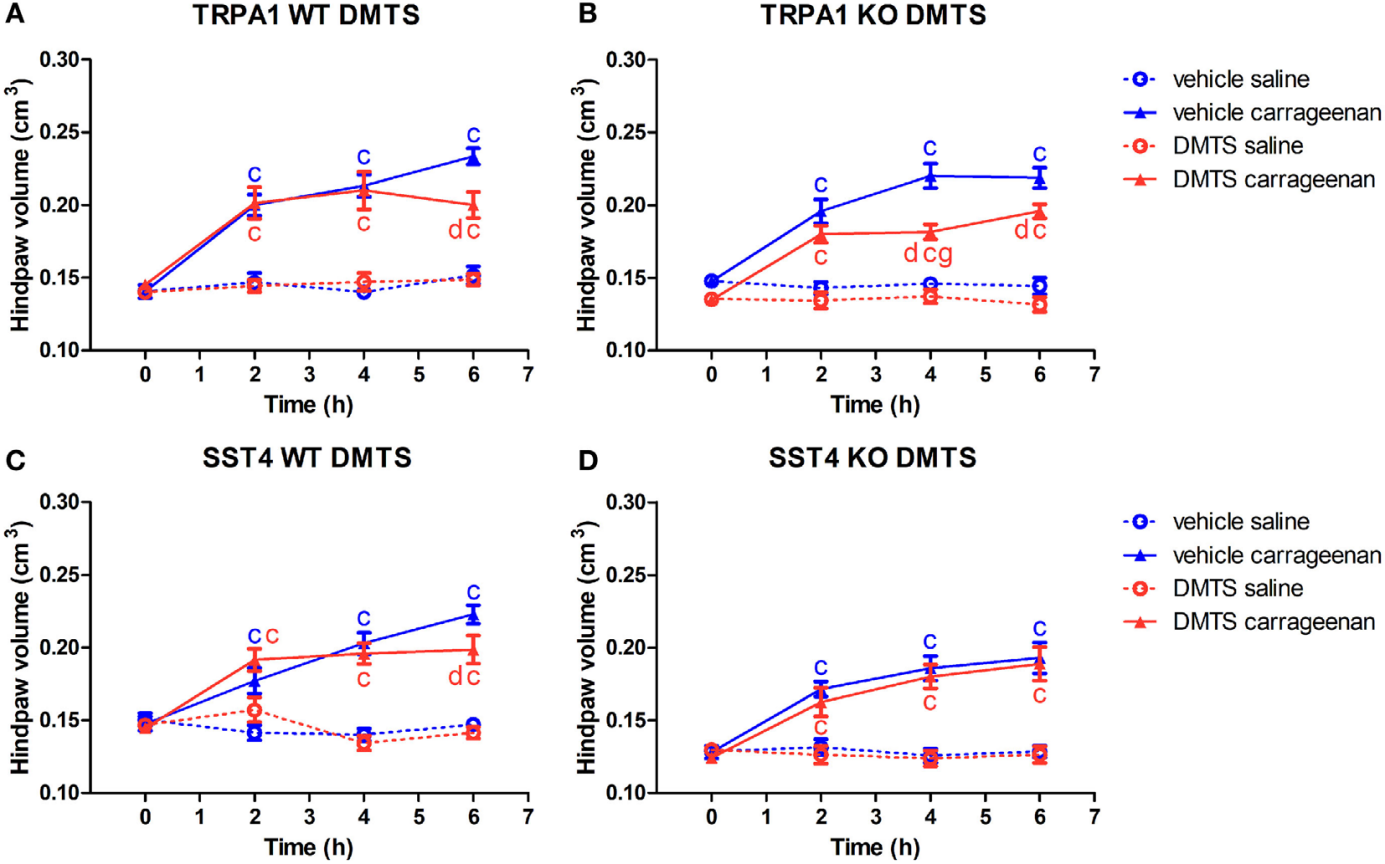
Alleviating effect of dimethyl trisulfide (DMTS, 250 µmol/kg, i.p.) on edema formation in carrageenan-induced hind paw inflammation is independent of the transient receptor potential ankyrin 1 (TRPA1) ion channel, but is mediated by somatostatin (SOM) sst4 receptors. Effect of DMTS or vehicle treatment on hind paw edema detected by plethysmometry in saline or carrageenan-treated (3% in 20 µL saline) feet of **(A)** TRPA1 WT, **(B)** TRPA1 KO, **(C)** sst4 receptor WT, and **(D)** sst4 receptor KO mice. Data are shown as mean ± SEM. *n* = 6–8. ^c^*p* < 0.05 vs. saline-injected paws. ^d^*p* < 0.05 vs. vehicle of DMTS. ^g^*p* < 0.05 vs. TRPA1 WT animals. Two-way repeated-measure ANOVA followed by Bonferroni’s multiple comparison test.

Transient receptor potential ankyrin 1 WT and KO mice developed significant swelling of the hind feet irrespectively of DMTS or vehicle treatment (*n* = 6–7). DMTS ameliorated swelling at 6 h in carrageenan-injected feet of TRPA1 WT mice compared to those of vehicle-treated ones (*n* = 6–7; Figure 4A). DMTS significantly relieved swelling in carrageenan-treated paws of TRPA1 KO mice at 4 and 6 h after challenge in comparison with those of vehicle-treated ones (*n* = 7; Figure 4B). DMTS produced a stronger inhibition of swelling in the carrageenan-injected feet of TRPA1 KO animals at 4 h than in those of TRPA1 WT mice (*n* = 7; Figure 4B). Edema formation in saline-injected feet of TRPA1 WT and KO mice was not affected by DMTS or vehicle treatment.

Carrageenan challenge lead to significant paw swelling in sst4 receptor WT and KO mice irrespectively of vehicle or DMTS treatment (*n* = 7–8). DMTS relieved edema formation in carrageenan-treated paws of sst4 WT animals at 6 h in comparison with those of vehicle-treated ones (*n* = 7; Figure 4C). DMTS did not show any protective effect in sst4 receptor KO mice (Figure 4D).

### Carrageenan-Evoked MPO Activity of Accumulated Neutrophil Cells Is Unaffected by Administration of POLY

**Figure 5:**
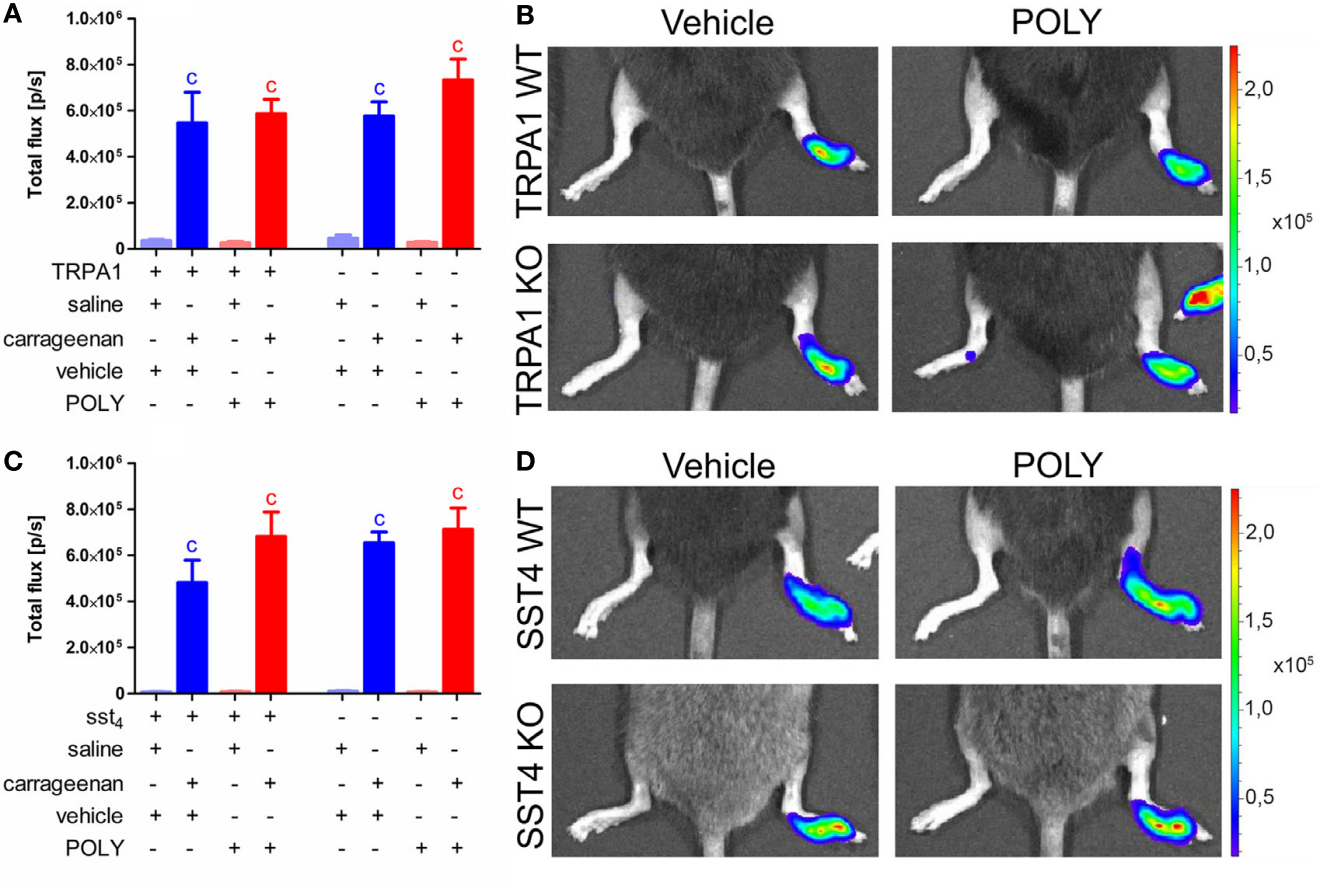
Polysulfide (POLY) treatment (17 µmol/kg, i.p.) does not alter myeloperoxidase (MPO) activity shown by luminol bioluminescence in murine hind paws with carrageenan-induced inflammation. **(A)** Bioluminescence in saline and carrageenan-injected (3% in 20 µL saline) hind feet of transient receptor potential ankyrin 1 (TRPA1) WT and KO animals. **(B)** Representative bioluminescent images of saline and carrageenan-treated (3% in 20 µL saline) hind paws of TRPA1 WT and KO mice illustrating MPO activity. **(C)** Luminol bioluminescence in saline and carrageenan-treated (3% in 20 µL saline) hind feet of sst4 receptor WT and KO mice. **(D)** Representative bioluminescent images of saline and carrageenan-treated (3% in 20 µL saline) hind paws of sst4 WT and KO animals. Data are shown as mean ± SEM. *n* = 7–8. ^c^*p* < 0.05 vs. saline-injected paws. One-way ANOVA followed by Bonferroni’s multiple comparison test.

Both TRPA1 WT and KO animals developed significantly elevated MPO activity in carrageenan-injected hind paws independently from vehicle or POLY administration (*n* = 7). POLY did not ameliorate MPO activity in any animal groups nor did it affect the values of saline-injected control paws (Figures 5A,B). Similar data were produced in sst4 receptor WT and KO mice (*n* = 7–8; Figures 5C,D). Fluorescent determination of plasma extravasation following measurement of MPO activity produced no significant difference in either POLY or DMTS treated groups of any genetic background. (Datasheet 1 in Supplementary Material).

### DMTS Inhibits MPO Activity of Accumulated Neutrophil Granulocytes Independently of sst4 Receptors

**Figure 6:**
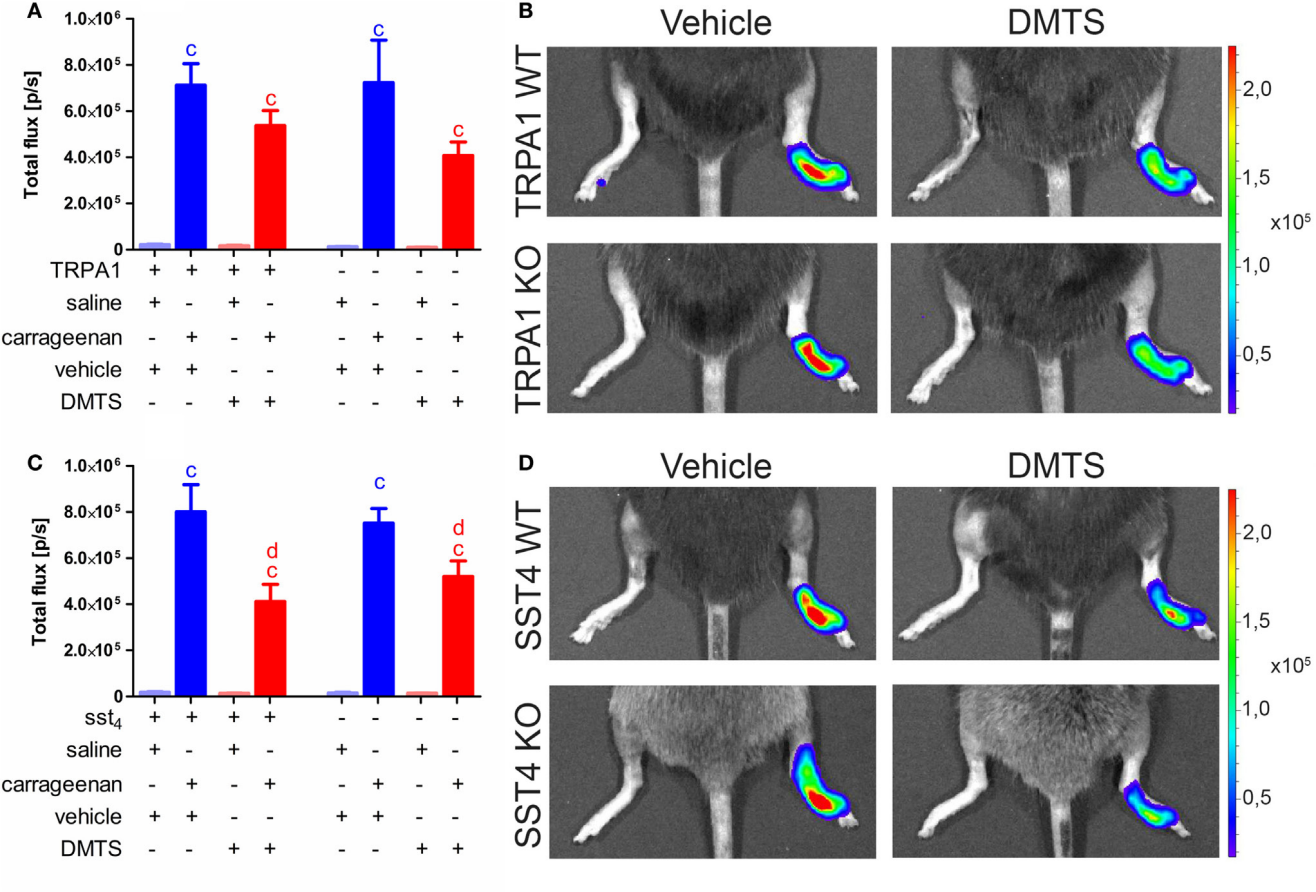
Dimethyl trisulfide (DMTS) administration (250 µmol/kg, i.p.) decreases myeloperoxidase (MPO) activity indicated by luminol bioluminescence in sst4 receptor WT and KO mice undergoing carrageenan-induced inflammation of the hind foot. **(A)** Bioluminescence in saline and carrageenan-treated (3% in 20 µL saline) hind paws of transient receptor potential ankyrin 1 (TRPA1) WT and KO animals. **(B)** Representative bioluminescent images of saline and carrageenan-treated (3% in 20 µL saline) hind paws of TRPA1 WT and KO mice depicting MPO activity. **(C)** Luminol bioluminescence in saline and carrageenan-treated (3% in 20 µL saline) hind paws of sst4 receptor WT and KO mice. **(D)** Representative bioluminescent images of saline and carrageenan-injected (3% in 20 µL saline) hind paws of sst4 WT and KO animals. Data are shown as mean ± SEM. *n* = 6–9. ^c^*p* < 0.05 vs. saline-injected paws. ^d^*p* < 0.05 vs. carrageenan-injected paws of mice treated with the vehicle of DMTS. One-way ANOVA followed by Bonferroni’s multiple comparison test.

Carrageenan-injected feet of TRPA1 WT and KO animals developed significantly increased MPO activity irrespectively of vehicle or DMTS administration (*n* = 6–7). MPO activity in carrageenan-injected hind paws of DMTS-treated TRPA1 WT and KO mice tends to be smaller than in those of vehicle-treated ones (Figures 6A,B). Sst4 WT and KO mice showed significantly elevated MPO activity upon carrageenan injection independently of vehicle or DMTS treatment (*n* = 7–9). DMTS did not alter MPO activity of saline-injected control paws. DMTS ameliorated MPO activity in carrageenan-treated feet of both sst4 WT and KO mice compared to those of vehicle-treated ones (*n* = 7–9; Figures 6C,D).

## Discussion

The main novel findings of the current study are the following:

- Antinociceptive effect of POLY in carrageenan-evoked paw inflammation depends on TRPA1 ion channel opening by the drug, release of SOM from the activated peptidergic sensory nerve fibers and subsequent activation of sst4 receptors, as the antihyperalgesic effect of POLY was absent in TRPA1 and sst4 KO mice.
- Organic trisulfide DMTS possessed an antinociceptive effect not only in TRPA1 and sst4 receptor WT animals, but also in TRPA1 KO ones. Protective activity was significantly weaker in TRPA1 KO mice than in WT ones. These findings imply target molecules of DMTS on sensory nerve endings other than TRPA1 leading to activation of the fibers and SOM release.
- Polysulfide administration exhibited no statistically significant effect on carrageenan-induced paw edema, unlike DMTS that ameliorated swelling in TRPA1 WT, KO, and sst4 receptor WT mice. These data point toward other mediators of DMTS effect than TRPA1 on peptidergic nociceptors.
- Polysulfide had no effect on MPO activity of the inflamed hind paws. Interestingly, DMTS significantly lowered MPO activity characterizing neutrophil accumulation in sst4 receptor WT and KO animals. A similar trend emerged in TRPA1 WT and KO animals not bestriding the limit of statistical significance. Our results indicate a mechanism of action of DMTS regarding MPO activity differing completely from the one suggested by data on mechanical nociception and paw swelling. Activation of TRPA1, release of SOM and its effect on sst4 receptors do not contribute to the inhibition of MPO activity by DMTS.

Most authors familiar with the area agree that activation of TRPA1 ion channels on nociceptor sensory nerve endings by H_2_S, POLYs, or other agonists is painful due to activation of the ascending pain pathway (17, 36, 37). We do not debate that TRPA1 receptor activation is acutely painful. Intraperitoneal administration of POLY and DMTS surely evoked abdominal discomfort in our experimental animals. However, it is not only well documented scientifically, but exploited clinically that activation of peptidergic primary sensory neurons mediates a later onset antinociceptive effect (we refer to the dermal patch Qutenza^®^ with high capsaicin content used in the therapy of neuropathic pain and relying on a different mechanism of action than that suggested for POLY and DMTS by the present work).

It was reported earlier that peptidergic sensory nerve endings release neuropeptides upon activation, among them SOM. Beside a population of nociceptors SOM is expressed in the central nervous system and peripheral tissues, too (23, 38). Treatment with TRPA1 receptor agonists or nociceptor activation by other means leads to SOM release from primary sensory neurons and the peptide reaches significant concentration in the bloodstream (9, 39–42). SOM exerts antinociceptive and anti-inflammatory effects at parts of the body distant from the site of release. These effects were shown to be mediated by somatostatin sst4 receptors (9, 25, 28, 40). Antinociceptive and anti-inflammatory SOM effects are obviated by somatostatin receptor antagonist, depletion of SOM from sensory nerves, an antibody catching the peptide and genetic lack of the sst4 receptor. On the other hand, sst4 receptor agonists induce similar beneficial effects to those of SOM (24, 30). Sst4 receptors expressed in sensory neurons, lymphocytes, and vascular endothelial cells might contribute to the protective effect (25). Non-neuronal sources of TRPA1 activation-induced surge of SOM in the circulation shall not be taken into account, hence denervation or defunctionalization of the area exposed to TRPA1 agonist prevented such effects (39, 43).

Somatostatin is a prerequisite of antihyperalgesic and anti-inflammatory effects mediated by peptidergic nerve endings. It is known that other mediators contribute too. The sensory neuron-dependent antinociceptive effect was abolished by antagonism of opioid receptors. Opioid peptides might be released from sensory neurons and leukocytes (39).

According to our data activation routes of the sensory neuron–somatostatin axis other than TRPA1 ion channels are in play in case of DMTS, as the organic trisulfide elicited antinociceptive effect and inhibited paw swelling independently of TRPA1, but still *via* sst4 receptors. Similar mechanisms might have been in play leading to the trend of inhibition of hind paw edema detected by plethysmometry in TRPA1 KO mice treated with POLY (Figure 3B). Several such mechanisms were suggested for H_2_S. TRPV1 channels co-expressed with TRPA1 can be ruled out because DMTS failed to produce Ca^2+^ signals in CHO cells expressing the channel (9). Taken into account that organic trisulfides are donors of H_2_S, these mechanisms might be valid for DMTS as well (10). Conversion of inorganic POLY into sulfide in living cells is an active field of research and remains to be elucidated. H_2_S was reported to activate T-type CaV 3.2 channels of sensory neurons (36). These ion channels modulate pain sensation by regulating the activity of sensory neurons (44). It has to be noted that inhibition of CaV 3.2 channels by H_2_S was detected, too. Supraphysiological concentration of H_2_S behaves rather as an activator, while normal concentration leads to inhibition of T-type Ca^2+^ channels (45). Voltage-gated K^+^ channels are potential mediators of the effects of DMTS too. KV 4.3 voltage-gated K^+^ channels are expressed in DRG neurons (46). H_2_S was reported to contract murine gastric smooth muscle by persulfidation of KV 4.3 channels. Inhibition of KV 4.3 channels was reproducible in H293 cells and could be diminished by a reducing agent and a blocker of free thiol groups that prevent protein persulfidation (47). Ability of the organic trisulfide DMTS to inhibit voltage-gated K^+^ channels could contribute to depolarization of peptidergic sensory neurons and SOM release from these cells.

Sodium POLY is an anionic compound, thus it most probably cannot penetrate into the central nervous system. It reacts readily with cysteine amino acids of proteins and loses its negative charge. However, proteins are excluded from the brain and cannot transport POLY there. This way the effects of POLY described in the present study might rely on a peripheral mechanism (even SOM released from the sensory nerves is excluded from the central nervous system). Potassium POLY was found to enter intact HEK293T cells and produce protein persulfidation (6). Organic trisulfides such as DMTS are highly lipophilic and penetrate the blood–brain barrier freely. An uptake *via* facilitated diffusion or active transport has been proposed in case of DMTS too (48). Target proteins in the spinal cord and brain available for DMTS might contribute to its differing effect on nociception from that of POLY.

Mechanical pain threshold data of carrageenan-injected feet of TRPA1 and sst4 WT and KO animals treated with vehicle of POLY or DMTS were analyzed by one-way ANOVA followed by Tukey’s test. Statistically significant difference was found between POLY- and DMTS-treated TRPA1 WT mice at 2 h (*p* < 0.05), POLY- and DMTS-treated sst4 WT animals at 0 (*p* < 0.05) and 6 h (*p* < 0.01), POLY- and DMTS-treated sst4 KO mice at 0 (*p* < 0.05) and 6 h (*p* < 0.01). It is needless to state that it makes no sense to compare TRPA1 and sst4 strains. The above differences do not influence the power of conclusions on the mechanism of either DMTS or POLY action because conclusions were drawn from within either POLY- or DMTS-treated groups, where influencing factors were homogeneous.

Interestingly, a smaller paw volume was detected at 4 and 6 h in the carrageenan-injected hind paws of POLY-treated sst4 KO mice compared to the WT ones. This might conflict with protective nature of SOM discussed above. Compensatory changes in the expression of inflammatory genes in knockout animals might be responsible. Unfortunately, the sst4 receptor gene-modified mouse strain utilized in the present study has not been characterized yet in that regard. However, similar results were published on another protective neuropeptide and its receptor: pituitary adenylate cyclase-activating polypeptide (PACAP) and VPAC1 receptor. PACAP is usually known as a protective peptide. Experimental autoimmune encephalomyelitis (EAE) was found to be more severe in PACAP peptide knockout mice (49). Mirroring our findings on sst4 SOM receptors, animals genetically lacking VPAC1 PACAP receptors exhibited ameliorated responses in the same EAE model and in dextran sulfate-evoked colonic inflammation too (50, 51). VPAC1 KO mice had decreased mRNA levels of T_h2_ cytokines and chemokines (50). A similar compensatory mechanism in sst4 KO animals might influence the activation of neutrophils and macrophages—the dominant cells involved in carrageenan-induced paw edema inflammation—and decrease edema formation (52).

An inhibitory effect of DMTS on MPO activity was found that is mediated by neither TRPA1 nor SOM. Sulfide potentially being released from DMTS directly inhibits MPO activity of neutrophil granulocytes offering a straightforward mechanism (10, 53). Sulfide was documented to inhibit neutrophil cell accumulation and formation of reactive oxygen species in murine ventilator-induced lung injury, as well as to interfere with Ca^2+^-dependent cytoskeletal activities (chemotaxis and release of azurophilic granules) of human neutrophils (54, 55). H_2_S suppressed adherence of rat neutrophil granulocytes in the mesenteric blood vessels detected by intravital microscopy. The effect was found to be mediated by the inhibition of K_ATP_ ion channels (56, 57). Similarly, recruitment of human neutrophil cells was reduced by sulfide by the stimulation of L-selectin shedding. L-selectin is necessary for the adhesion of the inflammatory cells to endothelium. Activation of TNF-α-converting enzyme (ADAM-17) is supposed to be responsible (58).

We conclude that activation of peptidergic sensory neurons, release of SOM and subsequent activation of sst4 receptors are important mediators of antihyperalgesic effect of both POLY and DMTS. Unlike POLY, DMTS possesses anti-inflammatory activity too. The aforementioned mechanism contributes to the amelioration of edema formation by DMTS complemented by other means of peptidergic-nerve activation as the effect depends on the presence of functional sst4 receptors. DMTS is able to suppress MPO activity of neutrophil granulocytes at the site of inflammation without the involvement of the sensory neuron–SOM axis. Superior chemical stability, favorable pharmacokinetic properties, and significant translational potential—due to being a recognized food additive and having been patented as cyanide antidote—set DMTS in front of sodium POLY as a candidate of drug development which is only set back by the characteristic odor of the substance.

## Ethics Statement

All experimental procedures were carried out according to the European Communities Council Directive of 2010/63/EU. The studies were approved by the Ethics Committee on Animal Research, University of Pécs (license number: BA02/2000-47/2017).

## Author Contributions

IB designed and performed *in vivo* experiments, as well as fluorescent and luminescent imaging. He was involved in sodium polysulfide synthesis and cold cyanolysis and evaluation of data. HÁ performed fluorescent and luminescent imaging. HZ and PE contributed to the conception and design of the study and provided financial background. PG designed the study, performed *in vivo* experiments, contributed to sodium polysulfide synthesis, and drafted the manuscript. He provided funding too.

## Conflict of Interest Statement

The authors declare that the research was conducted in the absence of any commercial or financial relationships that could be construed as a potential conflict of interest.
