# Supplementary material for: Role of Transient Receptor Potential Ankyrin 1 Ion Channel and Somatostatin sst4 Receptor in the Antinociceptive and Anti-inflammatory Effects of Sodium Polysulfide and Dimethyl Trisulfide
Source: Front Endocrinol (Lausanne). 2018 Feb 27;9:55. doi: 10.3389/fendo.2018.00055 (PMC5835328; doi:10.3389/fendo.2018.00055)
Supplement: Supplementary file 1 [file Data_Sheet_1.DOCX]

Supplementary Material

Role of TRPA1 ion channel and somatostatin sst4 receptor in the antinociceptive and anti-inflammatory effects of sodium polysulfide and dimethyl trisulfide

Bátai István Z., Horváth Ádám, Pintér Erika, Helyes Zsuzsanna, Pozsgai Gábor^*^

*** Correspondence:** [gabor.pozsgai@aok.pte.hu](mailto:gabor.pozsgai@aok.pte.hu)

Department of Pharmacology and Pharmacotherapy

Medical School

University of Pécs

Szigeti u. 12.

Pécs

H-7624

Tel.: +3672-536-000/31642

Fax: +3672-536-218

# Supplementary Data

- 1. **Detection of plasma extravasation in the hind paws by fluorescent imaging**

Animals were anaesthetized with ketamine and xylazine (120 mg/kg and 12 mg/kg) 6 hours after hind paw challenge. Fluorescent dye IR-676 (0.5 mg/kg) in Kolliphor HS 15 (5% v/v) micelles was administered into the retrobulbar venous plexus. The size of micelles enables extravasation of the dye only in case of pathologically increased vascular permeability (1). Fluorescence of IR-676 was captured 20 min after administration. Measurements were conducted in an IVIS Lumina II (PerkinElmer, Waltham, USA; auto acquisition time, F/stop=1, Binning=2, excitation/emission filter: 640/700 nm) instrument and Living Image® software (Perkin-Elmer, Waltham, USA). Identical regions of interest (ROIs) were applied to both hind paws and calibrated units of fluorescence (total radiant efficiency = (photons · cm^2^)/(s · µW) originating from the ROIs were detected (1).

- 1. **POLY treatment does not influence carrageenan-induced plasma extravasation detected by fluorescent imaging**

Neither TRPA1 WT nor KO mice exhibited statistically significant plasma extravasation in their carrageenan-injected hind paws irrespectively of vehicle or POLY treatment (n = 7). POLY did not show any protective effect in TRPA1 WT or KO animals regarding carrageenan-evoked plasma extravasation nor did it change the process in saline-treated feet (Suppl. fig. 1A, B). Similar findings were obtained in sst4 receptor WT and KO mice (n = 7-8; Suppl. fig. 1C, D).

- 1. **Treatment with DMTS does not alter carrageenan-induced plasma extravasation in hind paws of mice**

Carrageenan injections resulted in significant plasma extravasation in the hind feet of both DMTS and vehicle treated TRPA1 WT animals (n = 6-7; Suppl. fig. 2A, B). Similar phenomenon occurred in carrageenan-injected paws of vehicle-treated sst4 WT and KO mice (n = 5-8; Suppl. fig. 2C, D). DMTS treatment did not lead to statistically significant changes in any animal groups.

- 1. **Discussion**

Despite inhibition of paw swelling by DMTS, plasma extravasation in inflamed hind feet detected by fluorescent imaging was unaffected by both POLY and DMTS treatments. It might produce a contradictory impression that in our hands DMTS alleviated swelling of inflamed hind paws, but failed to inhibit extravasation of micellated IR-676 fluorescent dye. The two methods provide information on different aspects of edema formation. Paw swelling measured as the volume of the paw by plethysmometry gives a picture of the equilibrium of plasma extravasation and resolution that occurred from the initiation of inflammation until the measurement. Fluorescence of IR-676 is captured after 20 min of i.v. administration. This way fluorescent imaging characterizes plasma extravasation during this short period of time. Our plethysmometry data describe the net extravasated plasma volume that had been produced along 2, 4 or 6 hours since the initiation of paw inflammation with carrageenan. They provide an overview of a relatively “chronic” process. Data generated with fluorescent imaging rather show the actual rate of plasma extravasation in the last 20 min after the injection of the fluorescent marker. These results characterize a more “acute” aspect of edema formation. Our negative results gained with the fluorescent marker IR-676 denote that the actual inhibition of plasma extravasation by DMTS is not present during the last 20 min of the 6 h inflammation. Based on plethysmometry data, this effect develops between 2 and 6 h after carrageenan challenge. A fluorescent test performed earlier (around 4 or 5 h after challenge) could have detected a difference between vehicle and DMTS-treated animals more easily. It has to be pointed out that DMTS is administered repeatedly. Elimination half life of DMTS is app. 30-35 min in the blood, but disulfide and persulfide modifications might accumulate on thiol residues of proteins responsible for the biological effect (2,3).


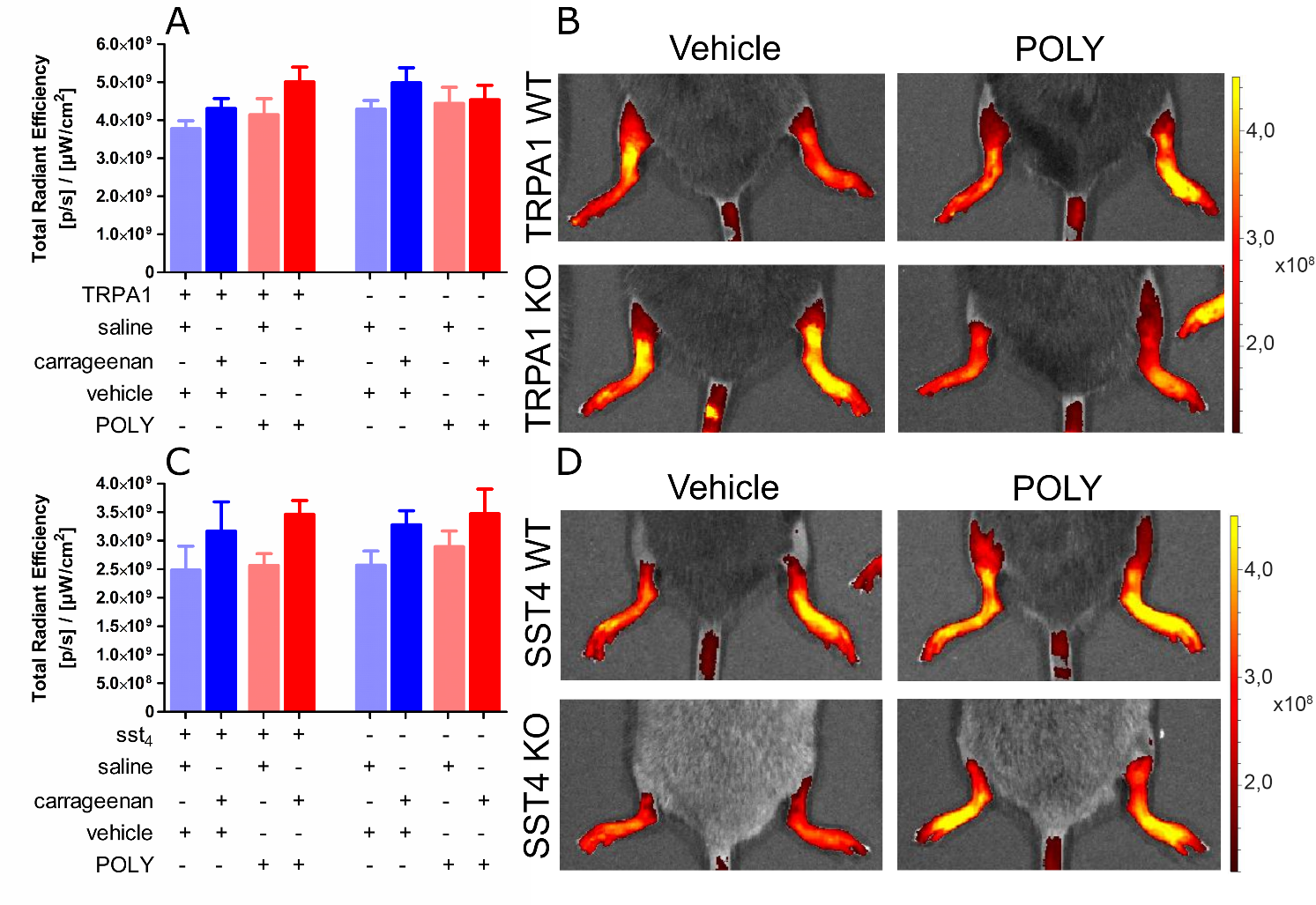


**Supplementary Figure 1.** Treatment with sodium polysulfide (POLY, 17 µmol/kg, i.p.) does not influence plasma extravasation of hind paws detected with luminescent imaging in the carrageenan model. (A) Fluorescence of micellated IR-676 dye in saline and carrageenan-injected (3% in 20 µL saline) hind feet of TRPA1 WT and KO mice characterizing plasma extravasation. (B) Representative fluorescent images of saline and carrageenan-treated (3% in 20 µL saline) hind paws of TRPA1 WT and KO animals depicting plasma extravasation. (C) Plasma extravasation shown by IR-676 fluorescence in saline and carrageenan-treated (3% in 20 µL saline) hind feet of sst4 receptor WT and KO mice. (D) Representative fluorescent images illustrating plasma extravasation in saline and carrageenan-injected (3% in 20 µL saline) hind feet of sst4 WT and KO animals. Data are shown as mean ± SEM. n = 7-8.


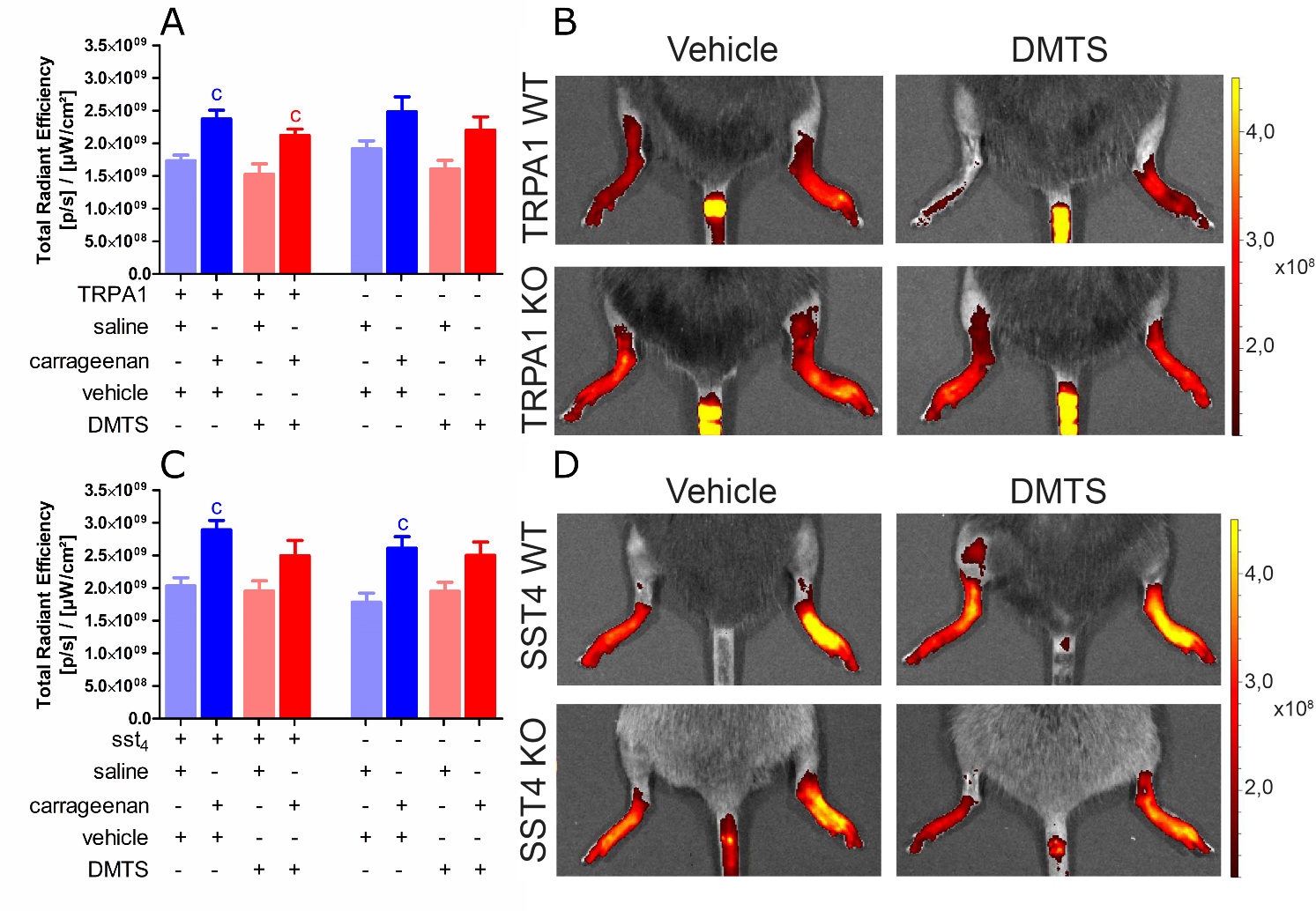


**Supplementary Figure 2.** DMTS administration (250 µmol/kg, i.p.) does not affect plasma extravasation detected as fluorescence of micellated IR-676 dye in hind paws of mice undergoing carrageenan-induced inflammation. (A) Fluorescence of IR-676 dye in saline and carrageenan-treated (3% in 20 µL saline) hind paws of TRPA1 WT and KO animals. (B) Representative fluorescent images of saline and carrageenan-injected (3% in 20 µL saline) hind feet of TRPA1 WT and KO animals showing plasma extravasation. (C) Plasma extravasation characterized by IR-676 fluorescence in saline and carrageenan-treated (3% in 20 µL saline) hind paws of sst4 receptor WT and KO mice. (D) Representative fluorescent images depicting plasma extravasation in saline and carrageenan-injected (3% in 20 µL saline) hind feet of sst4 WT and KO animals. Data are shown as mean ± SEM. n = 6-8. c p < 0.05 vs. saline-injected paws. One-way ANOVA followed by Bonferroni’s multiple comparison test.

**References**

1. Borbély É, Botz B, Bölcskei K, Kenyér T, Kereskai L, Kiss T, et al. Capsaicin-sensitive sensory nerves exert complex regulatory functions in the serum-transfer mouse model of autoimmune arthritis. Brain Behav Immun [Internet]. Elsevier; 2015 Mar [cited 2017 Aug 24];45:50–9. Available from: http://www.ncbi.nlm.nih.gov/pubmed/25524130

2. De Silva D, Lee S, Duke A, Angalakurthi S, Chou C-E, Ebrahimpour A, et al. Intravascular Residence Time Determination for the Cyanide Antidote Dimethyl Trisulfide in Rat by Using Liquid-Liquid Extraction Coupled with High Performance Liquid Chromatography. J Anal Methods Chem [Internet]. Hindawi; 2016 [cited 2017 Oct 27];2016:6546475. Available from: https://www.hindawi.com/journals/jamc/2016/6546475/

3. Greiner R, Pálinkás Z, Bäsell K, Becher D, Antelmann H, Nagy P, et al. Polysulfides link H2S to protein thiol oxidation. Antioxid Redox Signal [Internet]. Mary Ann Liebert, Inc.; 2013 Nov 20 [cited 2017 May 1];19(15):1749–65. Available from: http://www.ncbi.nlm.nih.gov/pubmed/23646934
